# Dairy product consumption and hypertension risk in a prospective French cohort of women

**DOI:** 10.1186/s12937-020-0527-2

**Published:** 2020-02-05

**Authors:** Paola Villaverde, Martin Lajous, Conor-James MacDonald, Guy Fagherazzi, Marie-Christine Boutron-Ruault, Fabrice Bonnet

**Affiliations:** 1 Center for Research on Population Health, INSP (Instituto Nacional de Salud Pública), Cuernavaca, Mexico; 2 Université Paris-Saclay, Villejuif, France; 3 Université Paris-Sud, Villejuif, France; 4 Center for Research in Epidemiology and Population Health (CESP), Institut Gustave Roussy, INSERM (Institut National de la Santé et de la Recherche Médicale) U1018, Villejuif, France; 5 Department of Global Health and Population, Harvard T.H. Chan School of Public Health, Boston, MA United States of America; 6 Department of Population Health, Luxembourg Institute of Health, Strassen, Luxembourg; 7 Groupe hospitalier Paris St-Joseph, Paris, France

**Keywords:** Dairy products, Processed cheese, Yoghurt, Hypertension, Women, Epidemiology, Prospective studies

## Abstract

**Background:**

Among potentially modifiable factors, dairy product consumption has been inconsistently associated with hypertension risk. The objective of this study was to investigate the relation between dairy product consumption and the risk of hypertension among middle-aged women.

**Methods:**

In a prospective cohort of 40,526 French women, there were 9340 new cases of hypertension after an average 12.2 years of follow up. Consumptions of milk, yogurt, and types of cheese were assessed at baseline using a validated dietary questionnaire. Hazard ratios (HRs) and 95% confidence intervals (95% CI) for hypertension were estimated with multivariate Cox models with age as the time scale.

**Results:**

The mean dairy consumption was 2.2 + 1.2 servings/day, as cottage cheese (0.2 + 0.2 servings/day), yogurt (0.6 + 0.5 servings/day), milk (0.4 + 0.7 servings/day), and cheese (1.1 + 0.8 servings/day). There was no association between risk of hypertension and total dairy consumption (multivariate HR for the fifth vs. first quintile HR_5vs.1_ = 0.97 [0.91; 1.04]). There was no association with any specific type of dairy, except for a positive association between processed cheese consumption and hypertension (multivariate HR_4vs.1 =_ 1.12 [1.06; 1.18]; p trend = < 0.003).

**Conclusions:**

In this large prospective cohort of French women, overall consumption of dairy products was not associated with the risk of hypertension. Results regarding processed cheese must be further confirmed.

## Introduction

Dairy products are the main source of highly bioavailable calcium, and they are largely consumed worldwide [1]. In the last years, a differential effect of dairy products according to their fat and sodium contents has been discussed [2]. In a meta-analysis of 9 US and European studies including over 15,000 hypertension cases, total dairy, low-fat dairy and milk were inversely and linearly associated with a lower risk of hypertension, but associations were less clear for other types of dairy products [3]. In a Mendelian randomization study, there was no association between the allele associated with lactase persistence and risk of hypertension [4]. However, as demonstrated in another Mendelian randomization study [5], lactase persistence was associated with milk intake but not with consumption of other dairy products. Associations of different types of dairy products with risk of hypertension have been studied in observational studies, with overall an inverse association, mostly with low-fat dairy while associations tended to be null with cheese or high-fat dairy [6]. However, other studies failed to find any association with dairy products [7]. Dairy products are a complex group of foods. Milk has a complex fat content, and also contains proteins of high biological value, and minerals, but no sodium [8]. Fermented products such as yogurt or cottage cheese contain biopeptides with angiotensin converting inhibitor effects [9], while cheese has an important vitamin and mineral, but also sodium content [10].

In France, where dairy products (especially cheese), are widely consumed, two studies [11, 12] have previously evaluated the association between blood pressure and dairy consumption. The cross-sectional French Monica Study reported an inverse association between dairy intake and blood pressure, especially in the case of overall high calcium intake [12]. In the prospective SUVIMAX study, dairy intake was inversely associated with systolic and diastolic blood pressure [11]. The aim of the present study was to investigate the relation between total dairy intake and different dairy products including types of cheese, and the risk of hypertension in a large cohort of French middle-aged women.

## Methodology

### The E3N cohort study

The E3N (Etude Epidémiologique de la Mutuelle Générale de l’Education Nationale (MGEN)) cohort study was set up in 1990, and included 98,995 women born between 1925 and 1950 and affiliated to the MGEN, a health insurance plan for workers in the education system and their spouses. The objective was to identify risk factors for cancer and chronic conditions in women. At baseline, women signed an informed consent form in accordance to the French National Commission for Data Protection and Privacy. This study is the French component of the European Prospective Investigation into Cancer (EPIC) [13].

Participants were asked every 2–3 years to complete self-administered questionnaires to provide and update medical events and lifestyle. Between 1993 and 1995, 74,520 participants answered the third follow-up questionnaire that included a validated self-administrated diet history questionnaire [14]. Answers to that questionnaire were considered baseline for the present study. We excluded women with unrealistic energy intake defined as extreme values for the ratio between energy intake and required energy (the 1st and 99th percentiles of the distribution in the population, *n* = 1381), no follow-up after baseline (*n* = 3641), women with cancer (*n* = 4253), cardiovascular disease (*n* = 451), or hypertension (*n* = 24,222) before baseline, and 50 participants who reported no dairy product consumption from any source. The final study population included 40,526 women.

### Dietary data and dairy product assessment

In 1993, a 208-item dietary history questionnaire was sent to all cohort women to evaluate the habitual diet of the previous year. Women were asked to answer questions about quantities and frequencies of consumption of food groups. Eleven possible responses were available, never or less than once a month; 1 to 3 times a month, and 1 to 7 times a week. A photo booklet was added to help estimate portion sizes [15]. The questionnaire included 34 items on dairy products: milk (whole, semi skimmed, skimmed, concentrated, or powdered); yogurt (unsweetened, flavored, or with fruit; whole-milk or skimmed; and with natural or artificial sweetener); cottage cheese (same as for yoghurt); and six types of cheese (i.e. soft, processed, blue, semi-hard, hard, and goat cheese). Consumptions of butter, cream, and ice cream were not included, considering butter and cream as added fats, and ice cream, which is not systematically dairy-based, as a dessert.

Dietary data was converted to energy and nutrient intakes multiplying the intake frequency by the serving size of each item. We used food composition from the French food composition table of the French Information Center on Food Quality [16]. The validity and reproducibility of the questionnaire was previously evaluated in a subsample of 120 women similar to the E3N study, correlation between diet history and 12 24 h recalls was 0.67 for all dairy products [14], 0.51 for cheese, 0.63 for yoghurt, 0.39 for cottage cheese and 0.12 for milk (due to a large number of non-consumers in the recall study).

We used weekly servings for each dairy item and we defined total dairy consumption as the sum of servings of milk, yogurt, cottage cheese, and other cheese. We estimated that an average portion of dairy provides about 300 mg of calcium. Thus, the serving sizes were calculated in order to obtain this amount of calcium; we considered as a portion 250 ml for milk, 175 g for yogurt, 250 mg for cottage cheese, 80 g for soft cheese, 120 g for processed cheese, 50 g for blue cheese, 40 g for semi-hard cheese, 30 g for hard cheese, and 190 g for goat cheese.

### Hypertension assessment

Participants reported diagnosis of hypertension, date of diagnosis, and use of antihypertensive treatments at baseline (1993 questionnaire) and in each of the follow-up questionnaires (1995, 1997, 2000, 2005, and 2008 questionnaires). The majority (69%) of participants reported both month and year of any diagnosis. For the 14% of cases who only reported the year of diagnosis, the month was imputed to June. The median time between the date of diagnosis and the date of response to the first questionnaire after diagnosis was 12 months*.* Thus, when the date of diagnosis was missing (17%), we assigned the date of diagnosis to be 12 months before the date hypertension was self-reported. In 2004, a claims database became available for all participants. For cases identified after 2004, we used either the self-reported date of diagnosis or the first date of drug reimbursement for anti-hypertensive medications [diuretics, b-blockers, calcium, and angiotensin-converting enzyme inhibitors (Anatomical Therapeutic Chemical Classification System codes C02, C03, C07,C08, and C09), with whatever happened first as the date of diagnosis. In addition, the claims database enabled us to evaluate the validity of self-reports. In women who were alive in January 2004 and up to their response to the last questionnaire in 2008, we observed a positive predictive value of 82% of self-reported hypertension when we compared the self-report to a drug reimbursement that corresponded to any of the previously specified codes. We previously evaluated the validity of self-reported hypertension using insurance claims data and found a positive predictive value of 82% [17].

### Covariate assessment

Whenever possible we used covariates from the questionnaire that just preceded the study baseline questionnaire. Age, family history of hypertension, body mass index (BMI), diabetes, treated hypercholesterolemia and smoking were self-reported. Age at each questionnaire was estimated using the date of birth and the date when the questionnaire was answered. BMI was calculated using self-reported height and weight, as weight divided by height squared (kg/m^2^). Anthropometric measurements were found to be valid in a previous study. Correlations between self-reported and measured anthropometry were 0.94 for weight and 0.84 for height [18]. Diabetes cases were validated using medical insurance information [19]. A woman was considered dyslipidemic if she declared both diagnosis of dyslipidemia and a specific treatment. Smoking status was considered as non-smoker, former smoker, and current smoker. The 1993 questionnaire included items on weekly hours spent walking, cycling, performing light and heavy household chores, and recreational activities (e.g., tennis and swimming) and the daily number of flights of stairs climbed. Weekly METs (metabolic equivalents) were estimated multiplying the reported activity duration by the yearly average METs for each item based on values from the Compendium of Physical Activities [20]. Education was self-reported; two categories were used: with high school diploma and without high school diploma. The dietary questionnaire (1993) was used to estimate the habitual intakes of energy, alcohol, fruit and vegetables, processed meat, sodium, magnesium, and potassium. A Western diet score was determined from dietary data using principal component analysis, as previously described [16].

### Statistical analysis

We categorized total dairy, yogurt, cottage cheese, other cheese, and milk consumption in quartiles. Cox models with age as the time scale were used to estimate HR and 95% confidence intervals (95% CI). Time at entry was the age at baseline (1993), exit time was the age of diagnosis of hypertension, death (dates of death were available from medical insurance), last follow-up, or the end of the follow-up period (June 25, 2008), whichever occurred first. The median value for each exposure category was used to estimate linear trends across categories and was included as a continuous variable in statistical models.

For each type of dairy product, we progressively adjusted for risk factors for hypertension that could be associated to dairy intake. The first model was only adjusted for energy excluding alcohol. The second model was additionally adjusted for family history of hypertension (no/yes), diabetes (no/yes), treated hypercholesterolemia (no/yes), smoking status (never, former, current), education (without high school diploma, with high school diploma), body mass index (< 25, 25–29.9, ≥30), and physical activity (Mets-h/week, continuous). The third model was further adjusted for dietary variables (alcohol (g/d), fruits and vegetables (g/d), processed meat (g/d). Statistical tests for interaction or effect moderation between total dairy intake and BMI were performed. When missing data, were less than 5% thus we imputed by the median or by the mode. In all models, all considered dairy variables were mutually adjusted for. Models for specific types of dairy were mutually adjusted for all other types of dairy.

Due to the large number of variables assessed, we adjusted the *p*-value for statistical significance using the method of Bonferroni, resulting in a *p* = 0.003. All statistical analyses were performed using SAS program version 9.4.

## Results

There were 9340 cases of incident hypertension after an average 12.2 years of follow up, and 493,309 person-years. The mean age (SD) of the participants was 51.6 (6.2). The median number of weekly servings of dairy products was 14.8 (7.8) overall, and 0.8 (4.6) for consumption of milk, 3.6 (3.3) for yogurt, 0.6 (1.5) for cottage cheese, and 6.4 (5.4) for cheese. The proportion of consumers was 99% for cheese, and 57% for milk. Regarding cheese, 27.8% of women consumed up to one serving, 34.0%, 1 to 2 servings, 21.2%, 2 to 3 servings, and 17.0%, at least four servings per day. Table 1 provides a description of the population per quartile of total weekly dairy intake. Compared with women in the first quintlie of dairy intake, women in the fifth quintile were slightly less often current smokers and hypercholesterolemic; they consumed more energy, more processed meat but also more fruit and vegetables, resulting in mode sodium, potassium and magnesium. Women consuming higher levels of processed cheese had lower levels of education than low-consumers.

**Table 1:** Population characteristics according to total dairy products consumption. E3N Cohort, 1993–2008 (*N* = 40,526)

Median weekly dairy servings (range)	Q1 (*N* = 8106) 7.1 (< 9.1)	Q2 (*N* = 8105) 11.1 (9.1–12.6)	Q3 (N = 8105) 14.3 (12.6–16.1)	Q4 (N = 8105) 18.3 (16.1–21.0)	Q5 (N = 8105) 25.5 (> 21.0)
Risk factors (mean (sd))					
Age (years)	51.8 (6.3)	51.7 **(**6.3)	51.6 (6.3)	51.4 (6.1)	51.5 (6.1)
Family history of hypertension (%)	28.8	29.0	28.3	29.7	27.7
Body mass index	22.0 (2.7)	22.1 **(**2.6)	22.2 **(**2.7)	22.4 **(**2.7)	22.5 (2.8)
Diabetes (%)	0.4	0.35	0.6	0.4	0.5
Hypercholesterolemia (%)	5.2	4.8	5.0	4.1	4.3
Smoking status (%)					
Never	51.1	50.9	52.6	52.6	52.4
Former	32.0	34.3	34.5	34.2	34.1
Current	16.9	14.8	12.9	13.2	13.5
Physical activity (Mets/week)	53.1 **(**30.0)	54.0 (29.4)	54.8 (29.6)	54.7 (29.9)	55.3 (30.0)
With high school diploma (%)	90.0	90.6	90.88	90.2	89.6
Dietary Factors (median (sd))					
Energy without alcohol (kcal/d)	1803.5 (453.3)	1953.3 (458.2)	2071.5 (474.7)	2196.4 **(**503.8)	2432.5 (557.7)
Alcohol (g/d)	7.4 (14.8)	7.2 (13.8)	7.2 (13.0)	6.9 (13.2)	6.2 (13.1)
Processed meat (g/d)	20.3 (18.2)	22.7 (18.6)	23.7 (19.0)	24.6 (20.4)	25.7 (23.2)
Fruits and vegetables (g/d)	560.5 (256.2)	599.2 (246.1)	625.5 (259.2)	648.9 (265.5)	681.3 (300.4)
Sodium (mg/d)	2275.3 (755.7)	2531.5 (750.2)	2716.7 (798.4)	2926.0 (844.4)	3311.1 (959.7)
Potassium (mg/d)	3257.8 (884.0)	3478.8 (881.3)	3691.9 (895.1)	3915.6 (951.0)	4342.2 (1129.3)
Magnesium (mg/d)	380.6 (133.3)	400.4 (130.3)	417.1 (133.6)	434.3 (139.1)	468.8 (153.3)
Milk (servings/week)	0 (1.0)	0 (1.3)	1.0 (1.4)	3.0 (1.6)	6.5 (1.9)
Cheese (servings/week)	3.4 (2.2)	5.2 (2.7)	6.7 (3.4)	8.4 (4.3)	12.0 (7.4)
Yoghurt (servings/week)	1.4 (1.8)	2.9 (2.5)	3.6 (2.8)	4.3 (3.1)	5.0 (4.1)
Cottage cheese (servings/week)	0.3 (1.0)	0.6 (1.3)	0.7 (1.4)	0.8 (1.6)	1.0 (1.9)
Mediterranean diet score	−0.12 (0.85)	−0.06 (0.88)	−0.01 (0.89)	0.02 (0.93)	0.08 (1.00)

In multivariable Cox models, there was no overall association between total dairy intake and hypertension risk (Table 2). When considering the various types of cheese (Table 3), there was no association with any type of cheese, except for consumption of processed cheese, which was associated with an increased risk of hypertension (HR for the third tertile of consumers vs. non-consumers: 1.12 [1.06; 1.18] p for trend < 0.003). There was no association between low-fat or high fat dairy, when considered separately, and risk of hypertension (Additional file 1: Table S1). When considering high-fat vs. low-fat yoghurt, regular consumption of high-fat yoghurt was associated with a slight increased risk of hypertension, while there was no association with low-fat yoghurt (Additional file 1: Table S2). Results were unchanged when replacing food covariates with a Mediterranean dietary score (not tabulated). No evidence for effect modification between dairy intake and BMI was observed.

**Table 2:** Dairy products consumption per day, and hypertension risk. E3N Cohort, France 1993–2008 (N = 40,526)

Dairy products (servings/week)	Cases	Person-years	M1		M2		M3	
			HR [95% CI]	p	HR [95% CI]	*p*	HR [95% CI]	*p*
Total dairy								
Q1 (< 9.1)	1858	98,273	Reference	0.38	Reference	0.41	Reference	0.86
Q2 (9.1–12.6)	1820	99,380	0.97 [0.90; 1.03]		0.97 [0.90; 1.03]		0.97 [0.91; 1.03]	
Q3 (12.6–16.1)	1890	98,609	1.01 [0.87; 1.04]		0.99 [0.93; 1.06]		1.00 [0.94; 1.07]	
Q4 (16.1–21.0)	1903	98,791	1.02 [0.85; 1.07]		0.99 [0.83; 1.06]		1.00 [0.94; 1.07]	
Q5 (> 21.0)	1868	98,257	0.99 [0.93: 1.07]		0.96 [0.89; 1.03]		0.97 [0.91; 1.04]	
Milk								
Q1 (0)	4042	210,401	Reference	0.54	Reference	0.66	Reference	0.23
Q2 (0–2.1)	1630	91,719	0.93 [0.88; 0.98]		0.93 [0.87; 0.99]		0.95 [0.88; 1.03]	
Q3 (2.1–4.2)	1861	97,132	1.02 [0.96; 1.09]		0.98 [0.92; 1.05]		0.94 [0.90; 0.99]	
Q4 (> 4.2)	1807	94,057	0.99 [0.91; 1.07]		1.01 [0.94; 1.08]		0.96 [0.61; 1.01]	
Yogurt								
Q1 (0)	1206	57,794	Reference	0.04	Reference	0.10	Reference	0.09
Q2 (0–2.9)	3078	169,861	0.92 [0.85; 0.98]		0.93 [0.87; 1.00]		0.99 [0.94; 1.05]	
Q3 (2.9–5.0)	2550	137,645	0.94 [0.87; 1.01]		0.95 [0.88; 1.02]		0.99 [0.94; 1.05]	
Q4 (> 5.0)	2506	128,009	1.02 [0.94; 1.10]		1.02 [0.94; 1.10]		1.06 [1.00; 1.12]	
Cottage cheese								
Q1 (0)	2463	131,350	Reference	0.72	Reference	0.38	Reference	0.23
Q2 (0–0.6)	2256	119,169	1.05 [0.99; 1.11]		1.03 [0.97; 1.10]		1.03 [0.97; 1.09]	
Q3 (0.6–1.6)	2237	120,049	1.03 [0.97; 1.09]		1.02 [0.96; 1.08]		1.05 [0.99; 1.11]	
Q4 (> 1.6)	2384	122,742	1.03 [0.97; 1.10]		1.00 [0.94; 1.06]		0.98 [0.92; 1.05]	
Cheese								
Q1 (< 3.7)	2300	122,557	Reference	0.53	Reference	0.57	Reference	0.51
Q2 (3.7–6.4)	2344	123,830	1.04 [0.98; 1.10]		1.05 [0.99; 1.11]		1.02 [0.96; 1.08]	
Q3 (6.4–9.7)	2404	123,130	1.01 [1.01; 1.16]		1.09 [1.02; 1.17]		1.05 [0.99; 1.11]	
Q4 (> 9.7)	2292	123,792	0.94 [0.94; 1.12]		1.03 [0.95; 1.14]		0.98 [0.92; 1.05]	

M1: Adjusted for energy (energy without alcohol (kcal/d)),

M2: M1+ smoking status (never, former, current), education (without high school diploma, with high school diploma), family history of hypertension (no, yes), and physical activity (mets/d))

M3: M2+ alcohol (g/d)), processed meat(g/d)), fruits and vegetables(g/d))

**Table 3:** Types of cheeses in servings per day, and hypertension risk, mutually adjusted and adjusted for all other dairy sources. E3N Cohort, France 1993–2008 (*N* = 40,526)

Cheeses (servings/week)	Cases	Person-years	M1		M2		M3	
			HR [95% CI]	*p*	HR [95% CI]	*p*	HR [95% CI]	*p*
Soft cheese								
Q1 (< 0.4)	2330	122,759	Reference	0.54	Reference	0.68	Reference	0.65
Q2 (0.4–0.8)	2319	123,613	1.01 [0.94; 1.08]		0.98 [0.91; 1.05]		0.98 [0.92; 1.05]	
Q3 (0.8–1.4)	2372	122,459	1.07 [0.99; 1.15]		1.04 [0.97; 1.12]		1.03 [0.96; 1.11]	
Q4 (> 1.4)	2319	124,478	1.04 [0.96; 1.12]		1.00 [0.92; 1.07]		0.98 [0.91; 1.06]	
Processed cheese								
Q1 (0)	3982	223,391	Reference	**< 0.0001**	Reference	**< 0.0001**	Reference	**< 0.0001**
Q2 (0–0.3)	1769	91,166	1.07 [1.01; 1.14]		1.08 [1.01; 1.15]		1.06 [0.96; 1.18]	
Q3 (0.3–0.6)	1760	88,328	1.12 [1.06; 1.14]		1.11 [1.05; 1.18]		1.10 [1.04; 1.16]	
Q4 (> 0.6)	1829	90,364	1.14 [1.06; 1.19]		1.12 [1.06; 1.19]		1.12 [1.06; 1.18]	
Blue cheese								
Q1 (< 0.2)	2371	124,255	Reference	0.66	Reference	0.35	Reference	0.58
Q2 (0.2–0.7)	2290	122,223	0.97 [0.91; 1.04]		0.98 [0.91; 1.04]		0.96 [0.90; 1.03]	
Q3 (0.7–1.4)	2367	124,194	1.01 [0.94; 1.08]		1.01 [0.94; 1.08]		0.99 [0.94; 1.06]	
Q4 (> 1.4)	2312	122,637	1.02 [0.95; 1.09]		1.03 [0.96; 1.10]		1.01 [0.96; 1.09]	
Semi-hard cheese								
Q1 (< 0.6)	2385	123,455	Reference	0.11	Reference	0.21	Reference	0.19
Q2 (0.6–1.3)	2373	122,593	0.99 [0.91; 1.06]		1.00 [0.93; 1.07]		1.00 [0.93; 1.06]	
Q3 (1.3–2.4)	2273	123,473	0.91 [0.85; 0.98]		0.92 [0.85; 0.98]		0.91 [0.90; 0.98]	
Q4 (> 2.4)	2309	123,789	0.94 [0.88; 1.02]		0.95 [0.89; 1.03]		0.95 [0.89; 1.03]	
Hard cheese								
Q1 (< 1.4)	2314	122,959	Reference	0.83	Reference	0.97	Reference	0.64
Q2 (1.4–2.5)	2338	123,572	1.05 [0.97; 1.11]		1.05 [0.98; 1.12]		1.04 [0.98; 1.11]	
Q3 (2.5–4.1)	2431	122,831	1.11 [1.04; 1.20]		1.11 [1.04; 1.19]		1.10 [1.02; 1.18]	
Q4 (> 4.1)	2257	123,948	1.00 [0.93; 1.10]		1.01 [0.94; 1.10]		1.00 [0.92; 1.08]	
Goat cheese								
Q1 (< 0.1)	2407	123,340	Reference	0.07	Reference	0.20	Reference	0.34
Q2 (0.1–0.2)	2390	123,660	0.97 [0.90; 1.04]		1.01 [0.94; 1.08]		1.01 [0.94; 1.08]	
Q3 (0.2–0.4)	2235	122,097	0.97 [0.89; 0.95]		0.93 [0.87; 0.99]		0.93 [0.87; 1.00]	
Q4 (> 0.4)	2308	124,213	0.97 [0.86; 1.08]		0.96 [0.90; 1.03]		0.98 [0.91; 1.05]	

M1: Adjusted for energy (energy without alcohol (kcal/d)),

M2: M1+ smoking status (never, former, current), education (without high school diploma, with high school diploma), family history of hypertension (no, yes), and physical activity (mets/d))

M3: M2+ alcohol (g/d)), processed meat(g/d)), fruits and vegetables(g/d))

Bold indicates *p*-values reaching statistical significance (< 0.003)

## Discussion

In this large prospective French cohort of women, we found no association between dairy product consumption and risk of hypertension, either overall or considering the various types of dairies. There was only a weak association with processed cheese consumption.

Previous studies about the relation between dairy consumption and hypertension provided inconsistent results [6, 21, 22]. In the Rotterdam study [6], low fat cheese and milk intake was associated with reduced hypertension risk, but this was not observed for higher fat cheeses. In the CARDIA study [21], increasing consumption of milk and dairy desserts was associated with lower hypertension risk. Cheese intake demonstrated no significant relationship with hypertension. In the Women’s Health study [22], there was an inverse relationship between intakes of skimmed milk and the risk of hypertension in middle-aged and older women. In our study, we observed no relation between consumptions of milk, or non-processed cheese, and risk of hypertension. In a recent systematic review and meta-analysis, an inverse dose-response relationship between dairy consumption and risk of hypertension was reported, with a 5% risk reduction per 200 g of dairy [20]. However, it is hard to translate into real-life consumption because of the large heterogeneity of serving sizes between the various dairy products from 30 g for hard cheese to 250 g for milk, which is the reason why we chose to use servings instead of adding grams of dairies of different calcium and protein concentrations. A former meta-analysis focused on blood pressure reported a small risk reduction of risk of hypertension with low-fat but not high-fat dairy. They again considered risk associated with 200 g dairy without specifying how different dairy products could be consumed. They suggested that the inverse association could be attributed to the high calcium and potassium intakes through dairy products, although they also suggested potential confounding by substituting unhealthy foods or drinks by dairy especially milk.

There has been much interest about the potential health benefits associated with probiotics, especially those from fermented dairy products such as yoghurt, because they could be beneficial to the microbiota [23]. In the Nurses’ Health study there was an inverse association between yogurt consumption and risk of hypertension, but yoghurt consumers also consumed less refined carbohydrates, sugar sweetened beverages and processed meat. In a meta-analysis of 5 prospective cohorts from the US and the Netherlands, there was no association between overall fermented dairy or any specific type of fermented dairy product with hypertension risk [3].

In our study, only processed cheese was associated with an increased risk of hypertension. Such products are made from various melted cheeses cooked with other ingredients such as milk, cream, butter, and sugar. Compared with traditional cheese, they have a higher salt, fat and sugar content, but a lower content of protein, magnesium, and calcium [24]. It is possible that the higher salt and fat contents drive the associations observed. However, considering the low weekly consumption, this result could also be explained by residual confounding. Consumption of these products, especially by adults, can be considered a behavioral rather than a true nutritional factor; they are cheaper than traditional cheese and associated with lower socioeconomic level in this cohort, and possibly adverse lifestyle behaviors. However, the observed association deserves further studies in other settings, in order not to overlook a true deleterious effect on health.

### Strengths and limitations

Our study has several strengths including a prospective design, long follow-up, validated dietary questionnaire, large sample size, and large number of cases enabling sufficient statistical power. In addition, the study was performed in a population with a high intake of dairy, cheese in particular, and we considered a large variety of commonly consumed dairy products, which allowed us to investigate specific associations.

However, there were some limitations. The dietary assessment was conducted once at the beginning of the study, which is subject to random error possibly attenuating any association. The validation study demonstrated good reproducibility after 1 year, and diet is quite stable in this elderly population [14]. In addition, consistent associations have been reported over time using this questionnaire for investigating a large range of outcomes. However, the food frequency questionnaire may have been inadequate to assess milk intake, as is indicated by the relatively low correlation coefficient. Incident hypertension was identified by self-report, and the validation study [17] confirmed the quality of self-report, but some degree of misclassification is possible, resulting in possibly attenuating any association. Finally, despite adjusting for the major risk factor for hypertension, we cannot rule out residual confounding by some unmeasured or poorly measured factors.

## Conclusion

In conclusion, our findings in a large prospective cohort of French women do not support a major role of dairy product consumption on the risk of hypertension in women. Results regarding processed cheese must be further confirmed. Consumption of dairy products can be associated with dietary habits and other lifestyle factors such as sports, differentially according to the country or gender. Overall, our results, in a country with a wide range of dairy products and a rather high level of intake, do not support a major effect of dairy product consumption on the risk of hypertension.

## Supplementary information

**Additional file 1: Table S1.** Whole-fat and low-fat and dairy consumption and hypertension risk (*N* = 40,526). E3N Cohort, France 1993–2008. **Table S2.** Types of dairy in servings per day, and hypertension risk, mutually adjusted and adjusted for all other dairy sources. E3N Cohort, France 1993–2008 (*N* = 40,526). Low consumers represent those consuming less than the median and high consumers more than the median.

## Data Availability

The datasets used and/or analysed during the current study are available from the corresponding author on reasonable request.
