# Supplementary material for: Dairy product consumption and hypertension risk in a prospective French cohort of women
Source: Nutr J. 2020 Feb 5;19:12. doi: 10.1186/s12937-020-0527-2 (PMC7003316; doi:10.1186/s12937-020-0527-2)
Supplement: Supplementary file 1 — Additional file 1: Table S1. Whole-fat and low-fat and dairy consumption and hypertension risk (N = 40,526). E3N Cohort, France 1993–2008. Table S2. Types of dairy in servings per day, and hypertension risk, mutually adjusted and adjusted for all other dairy sources. E3N Cohort, France 1993–2008 (N = 40,526). Low consumers represent those consuming less than the median and high consumers more than the median. [file 12937_2020_527_MOESM1_ESM.docx]

**Supplementary table 1. Whole-fat and low-fat and dairy consumption and hypertension risk (N= 40 526). E3N Cohort, France 1993 - 2008.**

| **Dairy products (servings)** | **Cases** | **Person-years** | **M1** | | **M2** | | **M3** | |
| --- | --- | --- | --- | --- | --- | --- | --- | --- |
|  |  |  | **HR [95% CI]** | **p** | **HR [95% CI]** | **p** | **HR [95% CI]** | **p** |
| High-fat |  |  |  |  |  |  |  |  |
| Q1 (0) | 2062 | 111401 | Reference | 0.02 | Reference | 0.27 | Reference | 0.09 |
| Q2 (0 - 0.6) | 1671 | 92380 | 0.99 [0.93; 1.06] |  | 0.99 [0.93; 1.05] |  | 0.99 [0.93; 1.05] |  |
| Q3 (0.7 - 1.6) | 1756 | 96681 | 0.99 [0.93; 1.05] |  | 0.97 [0.91; 1.03] |  | 0.97 [0.91; 1.03] |  |
| Q4 (1.7 - 3.6) | 1864 | 96510 | 1.03 [0.97; 1.10] |  | 1.00 [0.94; 1.07] |  | 1.01 [0.94; 1.07] |  |
| Q5 (> 3.6) | 1987 | 96338 | 1.09 [1.02; 1.16] |  | 1.04 [0.98; 1.11] |  | 1.06 [1.00; 1.13] |  |
| Low-fat |  |  |  |  |  |  |  |  |
| Q1 (0 – 1.1) | 2001 | 101223 | Reference | 0.97 | Reference | 0.71 | Reference | 0.76 |
| Q2 (1.2 – 3.7) | 1791 | 95861 | 0.97 [0.91; 1.03] |  | 0.96 [0.90; 1.03] |  | 0.96 [0.90; 1.02] |  |
| Q3 (3.8 – 6.0) | 1811 | 99012 | 0.94 [0.88; 1.00] |  | 0.94 [0.88; 1.00] |  | 0.95 [0.89; 1.01] |  |
| Q4 (6.1 – 9.8) | 1859 | 99027 | 0.98 [0.92; 1.04] |  | 0.97 [0.91; 1.03] |  | 0.98 [0.92; 1.04] |  |
| Q5 (>9.8) | 1878 | 98186 | 0.99 [0.93; 1.05] |  | 0.98 [0.92; 1.04] |  | 1.00 [0.93; 1.06] |  |

| M1: Adjusted for age and energy (energy without alcohol (kcal/d), |
| --- |
| M2: M1+ smoking status (never, former, current), education (without high school diploma, with high school diploma), family history of hypertension (no, yes), and  physical activity (Mets/d) |
| M3: M2+ alcohol (g/d), processed meat (g/d), and fruits and vegetables (g/d) |

**Supplementary table 2: Types of dairy in servings per day, and hypertension risk, mutually adjusted and adjusted for all other dairy sources. E3N Cohort, France 1993-2008 (N=40 526). Low consumers represent those consuming less than the median and high consumers more than the median.**

| **Dairy product** | **M1** | | **M2** | | **M3** | |
| --- | --- | --- | --- | --- | --- | --- |
|  | **HR [95% CI]** | **p** | **HR [95% CI]** | **p** | **HR [95% CI]** | **p** |
| **High fat yoghurt** |  |  |  |  |  |  |
| Non consumer (n = 31,099) | ref | **< 0.003** | ref | 0.02 | ref | **0.001** |
| Low consumer (n = 4,714) | 1.14 [1.07: 1.21] |  | 1.07 [1.00: 1.14] |  | 1.06 [1.00: 1.13] |  |
| High consumer (n = 4,713) | 1.15 [1.07: 1.23] |  | 1.06 [0.99: 1.14] |  | 1.09 [1.02: 1.16] |  |
| **Low fat yoghurt** |  |  |  |  |  |  |
| Non consumer (n = 9,566) | ref | 0.03 | ref | 0.06 | ref | 0.18 |
| Low consumer (n = 16,360) | 0.93 [0.88: 0.98] |  | 0.94 [0.89: 1.00] |  | 0.94 [0.89: 0.99] |  |
| High consumer (n = 14,600) | 0.93 [0.88: 0.99] |  | 0.94 [0.89: 1.00] |  | 0.95 [0.89: 1.00] |  |
| **High fat milk** |  |  |  |  |  |  |
| Non consumer (n = 37,988) | ref | 0.02 | ref | 0.19 | ref | 0.12 |
| Consumer (n = 2,538) | 0.90 [0.82: 0.98] |  | 0.93 [0.85: 1.01] |  | 0.93 [0.85: 1.01] |  |
| **Low fat milk** |  |  |  |  |  |  |
| Non consumer (n = 19,306) | ref | 0.90 | ref | 0.40 | ref | 0.58 |
| Low consumer (n = 11,105) | 0.97 [0.93: 1.02] |  | 0.96 [0.91: 1.00] |  | 0.96 [0.91: 1.03] |  |
| High consumer (n = 10,115) | 0.99 [0.95: 1.05] |  | 0.97 [0.93: 1.03] |  | 0.98 [0.93: 1.03] |  |
| **Sweetened yoghurt** |  |  |  |  |  |  |
| Non consumer (n = 8,992) | ref | **< 0.003** | ref | **< 0.003** | ref | 0.01 |
| Low consumer (n = 17,747) | 0.90 [0.86: 0.95] |  | 0.92 [0.87: 0.97] |  | 0.92 [0.87: 0.97] |  |
| High consumer (n = 13,787) | 0.89 [0.84: 0.94] |  | 0.90 [0.85: 0.95] |  | 0.92 [0.86: 0.97] |  |
| **Unsweetened yoghurt** |  |  |  |  |  |  |
| Non consumer (n = 26,354) | ref | 0.02 | ref | 0.006 | ref | 0.02 |
| Low consumer (n = 8,092) | 1.03 [0.97: 1.09] |  | 1.05 [0.99: 1.11] |  | 1.05 [0.99: 1.11] |  |
| High consumer (n = 6,080) | 1.07 [1.01: 1.14] |  | 1.08 [1.02: 1.15] |  | 1.08 [1.02: 1.15] |  |
|  |  |  |  |  |  |  |

| M1: Adjusted for energy (energy without alcohol (kcal/d)), |
| --- |
| M2: M1+ smoking status (never, former, current), education (without high school diploma, with high school diploma), family history of hypertension (no, yes), and physical activity(mets/d)) |
| M3: M2+ alcohol (g/d)), processed meat(g/d)), fruits and vegetables(g/d)) |
